# Key Challenges for Indigenous Peoples of Canada in terms of Oral Health Provision and Utilization: A Scoping Review

**DOI:** 10.1155/2022/7511213

**Published:** 2022-09-27

**Authors:** Ahmed Hussain

**Affiliations:** College of Dentistry, University of Saskatchewan, Health Science Building. Rm: E3332, 105 Wiggins Road, Saskatoon S7N 5E4, SK, Canada

## Abstract

**Background:**

The oral health of Indigenous peoples in Canada is lacking compared with their non-Indigenous counterparts. This scoping assessment aimed to investigate the obstacles of providing and using oral healthcare among Indigenous peoples in Canada.

**Methods:**

The scoping review took place between December 15, 2021 and January 10, 2022. Five key databases were examined: PubMed, Scopus, ISI Web of Science, Embase, and PROQUEST. The data were analyzed using NVIVO software to facilitate understanding of the major themes, subthemes, and codes provided.

**Results:**

Seven major themes and eighteen subthemes were identified as impacting the oral health provision and utilization of Indigenous peoples in Canada. The major themes are individual characteristics, affordability, availability, accessibility, accommodation, acceptability, and public or government policy. Thus, to improve the oral health of the Indigenous peoples in Canada, an integrated approach is required to address these obstacles.

**Conclusions:**

To address the oral health disparities among Indigenous peoples in Canada, policymakers should adopt an integrated approach.

## 1. Introduction

Oral health is important for good physical, mental, and psychological well-being [1]. Indigenous peoples have long been suffering from disparities in the Canadian healthcare system [2]. These disparities are even more evident in respect to the oral health of Indigenous peoples. Researchers used already collected data (such as the data from the Canadian Community Health Survey), questionnaires, and examinations to confirm these disparities [3–5]. Dental services have been provided to eligible First Nations and Inuit peoples through the Noninsured health benefits (NIHB) program. Different strategies have been implemented to eliminate these disparities, such as the First Nations oral health strategy teeth for life, the Inuit oral health action plan healthy teeth, healthy lives, British Colombia's First Nations and aboriginal oral health strategy healthy smiles for life [6–8]. However, the efforts implemented so far are not enough to bridge the gap between the oral health of Indigenous peoples of Canada and their nonindigenous counterparts.

Although the temporomandibular joint function is an integral part of the stomatognathic system [9, 10], no studies was published in this aspect related to the oral health of the Indigenous people of Canada.

From our initial scan of research articles published that identify obstacles in the provision and utilization of oral healthcare for Indigenous peoples of Canada, these articles have been limited to specific areas in Canada, specific physical or psychological characteristics, and may not reflect the current situation as they were published years ago. The intent of this scoping review was to thoroughly map the available literature regarding the challenges encountered by Indigenous peoples in achieving good oral health. It will allow us to identify and better understand knowledge gaps and focus on how these challenges could be addressed.

## 2. Methods

The approach was based on the five-stage system by Arksey and O'Malley with addition of a sixth stage presented by Levac et al. [11]. The approach is likewise supported by the Joanna Briggs Institute's approach for conducting scoping reviews [12]. Thus, the six stages included: (1) identifying the research question, (2) identifying relevant studies, (3) selecting studies, (4) charting the data, (5) collating, summarizing, and reporting the results, and (6) consultation with pertinent partners (optional) [11].

### 2.1. Identifying the Research Question

The following research question was used to determine the scope of the review and provide boundaries for the underlying search: ‘What are the key challenges for Indigenous people in Canada in terms of oral healthcare provision and utilization?'

### 2.2. Identifying the Relevant Studies

The research keywords/methodology was adopted from the study by Bastani et al.[13] with modifications. Five scientific databases were thoroughly searched (PUBMED, SCOPUS, PROQUEST, EMBASE, and Web of Science). In brief, with the support of an experienced librarian at the University of Saskatchewan, a precise search strategy was developed, utilizing particular MeSH terms [14] and keywords to gather pertinent material on the topic of interest. The Boolean concepts ‘OR,' ‘AND,' and ‘NOT' were combined to generate groups of keywords and medical subject titles. The search strategy was created for Medline using the Ovid interface (Table 1), and then adapted as necessary to enable a similar search on each of the other electronic platforms. Oral health, dental health, oral care, oral hygiene and delivery, provision, providing, utilization, use, access and challenges, problems, barriers, obstacles, Indigenous, native, aboriginal, First Nations, Metis, Inuit, and Canada were used in the search. Between the synonymous terms, the logical operator OR was utilized, and the logical operator AND was used to combine them.

The search strategy's eligibility criteria were created as per the PCC (Population-Concept-Context). Studies included in this review were those reporting on Indigenous people in Canada (population) oral health (concept) related to the obstacles of provision (context). Studies published in English language from January 1, 2000 to December 21, 2021 were included. Retained articles were original research articles (qualitative and quantitative).

**Figure 1:**
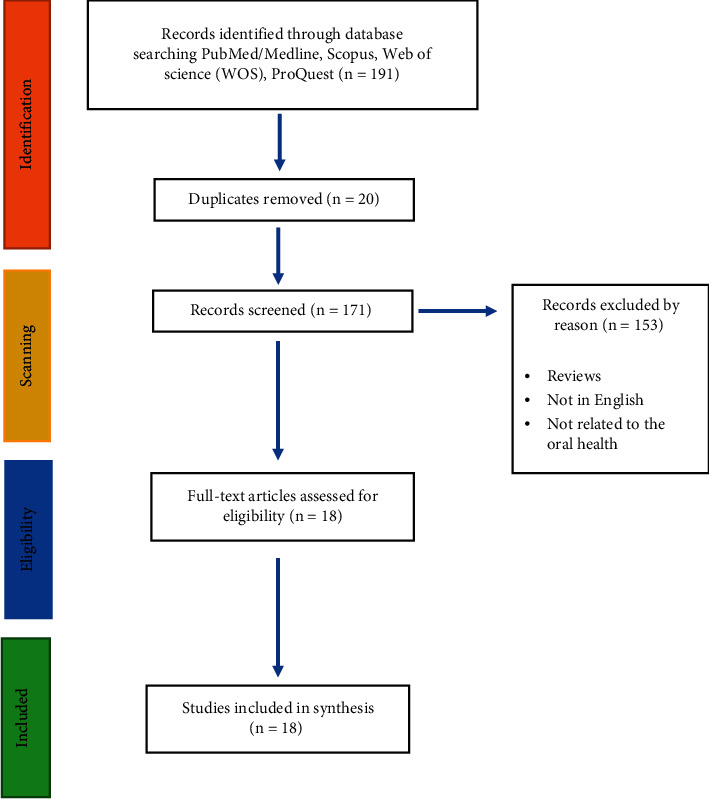
PRISMA flow diagram.

The exclusion criteria included study protocols, reviews, abstracts, opinions, editorials, letters, commentaries, and conference abstracts were excluded as this scoping review targeted peer-reviewed literature. Data collected prior to the date mentioned were excluded. Studies not originally published in English were excluded. The study selection and screening process is in accordance with the Preferred Reported Items in Systematic Reviews and Meta-Analyses (PRISMA) (Figure 1).

### 2.3. Selecting Studies

For this study, the screening process was as follows: the title and abstract of all retrieved citations (191 articles) were exported to an excel sheet. After removing duplicates and assessing the remaining articles against the inclusion criteria, 18 articles were included in the study. The method is summarized in a flowchart (Figure 1).

### 2.4. Charting the Data

The complete texts of the included articles were examined one-by-one, and the researcher used a data charting method (form) to extract the key study features. The author's name, year of publication, research title, study subject, study abstract, study design, and outcomes were included in the form.

### 2.5. Collating, Summarizing, and Reporting the Results

A summary (Table2) was generated of the included studies [11] to synthesize and summarize the findings. Qualitative descriptive analysis of the content was undertaken using NVivo V.12. The research questions were answered using a thematic analysis. We added to/modified the major themes published earlier in the Canadian Academy of Health Sciences (CAHS) report ‘*Improving access to oral health care for vulnerable people living in Canada*' [31]. The codes are categorized depending on how closely they are related and linked [32, 33].

## 3. Results

### 3.1. Study Selection

A total of 18 articles were used in the study (Table 2). For description, even though Indigenous peoples is the terminology presently in use in Canada, Table 2 retains the terminology used in the original article as some studies are from different locales or previous time periods.

### 3.2. Study Characteristics

**Figure 2:**
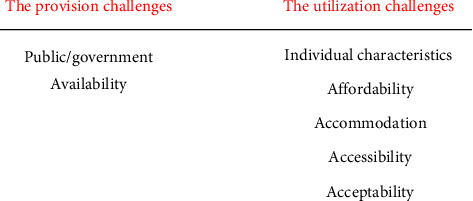
An overview of the scoping review's conceptual framework.

The CAHS outline from the report ‘*Improving access to oral health care for vulnerable people living in Canada*' report was used as a framework. However, we added to the outline as it was not inclusive of all the factors. There were 7 major themes and 18 subthemes that outline the challenges of oral health utilization by Indigenous peoples in Canada (Table 3).  Figure 2 shows a better understanding of the challenges that may be impacting the provision and utilization of oral healthcare.

### 3.3. The Impact of Individual Characteristics

Age, sex, medical status, psychological status, education, employment, oral health literacy, habits, lifestyle, and historical background were identified as the subthemes of the individual characteristic theme. These subthemes highlighted how Indigenous peoples' personal qualities might impact and shape oral healthcare utilization. In respect to age, young age or children are affected by early childhood caries (ECC), which can have long-lasting negative effects on their development [21, 25]. In addition, the elderly population seemed to experience more challenges in oral health [24, 34]. In respect to sex, in some studies [16] being a male is a risk factor, whereas in other studies being a woman, especially a pregnant woman [5], is a risk factor of having challenges with oral health. The link between poor physical/medical health and poor oral health has been pointed out [16]. The psychological status, such as the fear of dentist/needles and children being uncooperative, is a risk factor toward oral health utilization [25]. Having a higher education and employment were significantly related to better oral hygiene habits [26]. Limited oral health knowledge and habits such as smoking further reduce the quality of oral health of the individual [24, 26, 30, 35]. Insufficient knowledge of or inability to access healthy food choices is considered a barrier to good oral health [4, 20, 29]. Other factors such as poor access to clean water, fluoridated water, adequate sewage systems, electricity, or paved roads have affected oral health negatively [36]. Behaviors that do not support oral health and the gap between proper oral health knowledge and behavior have been mentioned as a limiting factor to achieving adequate oral health [24]. We should also keep in mind the shadows of historical factors (isolation, discrimination, and alienation) as a negative determinant to oral health [28].

### 3.4. The Impact of Affordability, Availability, and Accessibility

Affordability was represented as a major theme, with economic status as a subtheme, and encompasses lack of dental insurance, lack of affordability of oral hygiene supplies, and healthy food. Low income and scarce housing are risk factors for poor oral health experienced by Indigenous peoples in Canada [15, 17, 37]. Availability was a factor, which correlates to the lack of human resources, funding, and the uncertainty of many oral health programs [17, 36]. Accessibility also has an effect on oral health outcomes, and studies revealed that living in remote areas with no transportation affect the person's ability to access oral care services [26, 36].

### 3.5. The Impact of Accommodation, Acceptability, and Public/Government

Limitations in respect to the major theme of accommodation that compromise the provision of oral care include lack of financial incentive for the oral care provider and lack of accurate and complete health records [17, 28, 36]. This is further complicated by language barriers. Factors related to acceptability theme play a significant role in achieving good oral health. The lack of understanding/integration of the wholistic conceptualization causes a lack of community engagement [5, 17, 20]. Finally, the public/government as a major theme, with a policy subtheme shows a negative impact on oral healthcare due to low prioritization of oral health, budgetary constraints, and the policy gap between the federal and provincial powers [22]. Decision makers use the lack of scientific evidence about the effectiveness of certain modules to their hesitation to keep funding them [19].

## 4. Discussion

There are many obstacles and limitations faced by Indigenous peoples in Canada when it comes to accessing and utilizing oral healthcare. Earlier studies [36] identified four types of variables as barriers to oral health treatment among Indigenous peoples in Canada. These variables are affordability, accessibility, accommodation, and acceptability. Two more variables were added by our study—individual characteristics and public/government.

Individual characteristics, such as age (especially in children and the elderly), sex, medical status, psychological status, education, employment, oral health literacy level, habits, lifestyle, and behaviors, all have been found to reduce oral health usage among Indigenous peoples. This is in accordance with our previously published research where we found that age; gender; medical status; poor habits, such as smoking; limited level of education; and no employment resulted in poorer self-predicted oral health [3]. Bastani et al. reported similar findings for the Australian Indigenous population [13].

The economic status of Indigenous peoples has affected the affordability of oral health services. Low-income individuals cannot afford dental care, oral hygiene supplies, and healthy food. A similar pattern was reported in other parts of the world [38, 39].

The limited availability of oral health resources negatively impacted the oral health of the Indigenous peoples in Canada. For example, the lack of human resources on reserves in addition to the lack of funding for community programs and facilities led to the disruption of some dental services. Many programs operate on uncertain year-to-year funding cycles. Watt et al. reported a similar pattern, as he showed how resources facilitated access to oral healthcare [39].

Our results showed that living in remote areas, where the only oral health provider is miles away with no transportation services, significantly limits regular access to oral care. Such a barrier is clearly indicated in the First Nations” report [36].

The absence of financial incentive for pediatric residents, as an example, to do oral exams or apply fluoride is a limiting factor. Their busy schedules and a lack of accurate or complete medical records can make it even more challenging to provide oral healthcare [17]. The language barrier for some Indigenous peoples in Canada, as they do not speak English or French, has limited their access to oral health and made them more reliant on their community healers [34, 40].

Community acceptability plays an important role in integrating oral health programs. Racism and ignoring or undermining the traditional medical practice, through the lack of integration of the wholistic conceptualization of health, led to the lack of trust between the community and the oral health providers. This is because of the lack of proper training of the oral health providers [5, 34, 41].

Indigenous communities face numerous competing challenges, and this has led to low prioritization for oral healthcare. When there are budgetary constraints, oral healthcare programs often suffer the most. This is because of the lack of scientific research results that show the importance of these programs to the federal and provincial governments. National and local policymakers may consider designing and managing successful population-level initiatives to meet the oral health needs of all socially excluded communities, including the Indigenous population [13].

Given the multitude of factors that have been identified as having an effect on oral healthcare provision and utilization for Indigenous peoples in Canada, alternative approaches are necessary to bridge the gap and improve oral health outcomes. One approach is to provide oral care in an integrated form. Integrated care is emphasized as one of the basic concepts of primary care and defined as a coherent and coordinated set of services that are planned, managed, and delivered to individual service users across a range of organizations and by a range of cooperating professionals and informal carers [42].

### 4.1. Limitations

This review used previously published literature related to the oral health of Indigenous peoples in Canada. The number of studies included is not large. However, a similar pattern was recognized among medical health providers. We see a similar pattern in other parts of the world, such as in Australia.

## 5. Conclusion

Our findings suggest factors related to the individual characteristics, affordability, availability, accessibility, accommodation, acceptability, and public/government can limit Indigenous peoples' access to and provision of oral healthcare. Policymakers should look at addressing each one of these obstacles in a practical and interdisciplinary way to improve the oral health of the Indigenous peoples in Canada.

## Figures and Tables

**Table 1:** The search strategy of the scoping review.

#	Searches	Results
1	Oral health/	18632
2	Oral hygiene/	13452
3	Exp dentistry/or dental care/	420021
4	1 or 2 or 3	427544
5	Indians and North Americans/or exp indigenous Canadians/or Inuits/	17707

6	(Exp Indians and North American/or exp Inuits/or exp health services and Indigenous/or exp ethnopharmacology/or Athapaskan.mp. or Saulteaux.mp. or Wakashan.mp. or Cree.mp. or Dene.mp. or Inuit.mp. or Inuk.mp. or Inuvialuit^*∗*^.mp. or Haida.mp. or Ktunaxa.mp. or Tsimshian.mp. or Gitsxan.mp. or Nisga'a.mp. or Haisla.mp. or Heiltsuk.mp. or Oweenkeno.mp. or Kwakwaka'wakw.mp. or nuu chah nulth.mp. or Tsilhqot'in.mp. or Dakelh.mp. or Wet'suwet'en.mp. or Sekani.mp. or Dunne-za.mp. or Dene.mp. or Tahltan.mp. or Kaska.mp. or Tagish.mp. or Tutchone.mp. or Nuxalk.mp. or Salish.mp. or Stl'atlimc.mp. or Nlaka'pamux.mp. or Okanagan.mp. or sec wepmc.mp. or Tlingit.mp. or Anishinaabe.mp. or Blackfoot.mp. or Nakoda.mp. or Tasttine.mp. or tsuu T'inia.mp. or Gwich'in.mp. or Han.mp. or Tagish.mp. or Tutchone.mp. or Algonquin.mp. or Nipissing.mp. or Ojibwa.mp. or Potawatomi.mp. or Innu.mp. or Maliseet.mp. or Mi'kmaq.mp. or Micmac.mp. or Passamaquoddy.mp. or Haudenosaunee.mp. or Cayuga.mp. or Mohawk.mp. or Oneida.mp. or Onodaga.mp. or Seneca.mp. or Tuscarora.mp. or Wyandot.mp. or aboriginal^*∗*^.mp. or Indigenous^*∗*^.mp. or Métis.mp. or red road.mp. or “on-reserve”.mp. or off-reserve.mp. or First Nation.mp. or First Nations.mp. or amerindian.mp. or (urban adj3 (Indian^*∗*^ or native^*∗*^ or aboriginal^*∗*^)).mp. or ethnomedicine.mp. or country food^*∗*^.mp. or residential school^*∗*^.mp. or ((exp medicine, traditional/or traditional medicine^*∗*^.mp.) not Chinese.mp.) or exp shamanism/or shaman^*∗*^.mp. or traditional heal^*∗*^.mp. or traditional food^*∗*^.mp. or medicine man.mp. or medicine woman.mp. or autochtone^*∗*^.mp. or (native^*∗*^ adj1 (man or men or women or woman or boy^*∗*^ or girl^*∗*^ or adolescent^*∗*^ or youth or youths or person^*∗*^ or adult or people^*∗*^ or Indian^*∗*^ or nation or tribe^*∗*^ or tribal or band or bands)).mp.) and (exp Canada/or (Canada^*∗*^ or British Columbia or Colombie Britannique or Alberta or Saskatchewan or Manitoba or Ontario or Quebec or Nova Scotia or New Brunswick or newfoundland or Labrador or Prince Edward Island or Yukon territory or NWT or Northwest Territories or Nunavut or Nunavik or Nunatsiavut or NunatuKavut).mp.)	7322
7	5 or 6	20813
8	(Challenge^*∗*^ or obstacle^*∗*^ or problem^*∗*^ or barrier^*∗*^ or impediment^*∗*^ or hurdle^*∗*^ or hindrance^*∗*^).mp. (mp = title, abstract, original title, name of substance word, subject heading word, floating subheading word, keyword heading word, organism supplementary concept word, protocol supplementary concept word, rare disease supplementary concept word, unique identifier, and synonyms)	1864598
9	Exp “delivery of healthcare”/or health services accessibility/	1165064
10	8 or 9	2831483
11	Exp Canada/	172215

12	(Canad^*∗*^ or British Columbia or Alberta^*∗*^ or Saskatchewan or Manitoba^*∗*^ or Ontario or Quebec or (New Brunswick not New Jersey) or Nouveau Brunswick or Nova Scotia or Nouvelle Ecosse or Prince Edward island or newfoundland or Labrador or Nunavut or NWT or Northwest Territories or Yukon or Nunavik or Inuvialuit).mp, jw, nw. Or (Abbotsford or Airdrie or Ajax or Aurora or Barrie or Belleville or Blainville or Brampton or Brantford or Brossard or Burlington or Burnaby or Caledon or Calgary or Cape Breton or Chatham Kent or Chilliwack or Clarington or Coquitlam or Drummondville or Edmonton or Fredericton or Fort McMurray or Gatineau or Granby or Grande Prairie or Sudbury or Guelph or Halton Hills or Iqaluit or Inuvik or Kamloops or Kawartha Lakes or Kelowna or Kingston or Kitchener or Langley or Laval or Lethbridge or Levis or Longueuil or maple ridge or Markham or medicine hat or Milton or Mirabel or Mississauga or Moncton or Montreal or Nanaimo or New Westminster or Newmarket or Niagara Falls or Norfolk County or North Bay or North Vancouver or North Vancouver or Oakville or Oshawa or Ottawa or Peterborough or Pickering or Port Coquitlam or Prince George or Quebec city or red deer or Regina or Repentigny or Richmond or Richmond Hill or Saanich or Saguenay or Saint John or Saint-Hyacinthe or Saint-Jean-Sur-Richelieu or Saint-Jerome or Sarnia or Saskatoon or Sault Ste Marie or Sherbrooke or St Albert or St Catharines or St John's or Strathcona County or surrey or Terrebonne or Thunder Bay or Toronto or Trois-Rivieres or Vancouver or Vaughan or ((Cambridge or (Halifax or Hamilton or London or Victoria or Waterloo or Welland or Whitby or Windsor)) not (UK or Britain or United Kingdom or England or Australia)) or Whitehorse or Winnipeg or Wood Buffalo or Yellowknife) ti, ab, kw	452524
13	11 or 12	452526
14	4 and 7 and 10 and 13	67

**Table 2:** Characteristics of the included studies.

First authors and years	Study topics	Study methodologies	Conclusions
Kyoon-Achan et al. [15]	Challenges and problems faced by First Nations and Métis parents in meeting the early childhood oral health (ECOH) needs of their children in First Nations and Métis communities in Manitoba	Focus groups and sharing circles were conducted with four First Nations and Métis communities in urban and rural communities in Manitoba	Challenges identified included poor access to dental care, lack of transportation, lack of evidence-based oral health information to support good oral hygiene practices, experiencing poverty and food insecurity resulting in poor nutritional choices and leading to early childhood caries (ECC)

Mehra et al. [16]	Prevalence and factors associated with low dental-care utilization amongst Indigenous peoples in Ontario	Data from the 2014 cycle of the Canadian Community Health Survey was used	Factors identified included being male, a smoker, having fair/poor health, and lack of dental insurance

ElSalhy et al. [17]	Pediatric residents' perceptions of the feasibility of incorporating preventive dental care into a general pediatric outreach clinic for a First Nations community	Qualitative data was collected through focus groups using a semistructured interview guide	Challenges included that medical providers had limited knowledge on integrating oral health to pediatric care lack of knowledge and difficulty in applying the fluoride varnish, no financial incentive, access to care, no insurance for the patient, lack of accurate and complete records of patients

Shrivastava et al. [18]	Perspectives of patients, primary healthcare providers, and administrators at an indigenous healthcare organization regarding barriers and enablers of relational continuity of oral health care integrated within an indigenous primary healthcare organization	A multiple case study design within a qualitative approach and developmental evaluation methodology	Challenges identified were impermanence and lack of effective communication

Farmer et al. [19]	Dental hygiene perspectives on improving oral health outcomes in vulnerable populations in Canada	A qualitative study comprised of 16 one-on-one interviews conducted with dental hygienists between January and August 2015	Challenges included scarcity of evidence on interventions and their impact on oral health outcomes, including data availability from scientific research as well as mandatory reporting from institutions; logistical aspects related to the administration, structure, and sustainability (budgetary constraints within government) of programmes

Martin et al. [20]	Oral health perceptions of Inuit peoples and their dental service providers and exploring how differences might pose challenges and opportunities for oral health service delivery in NunatuKavut	Data collection included 18 qualitative focus groups (*n* = 108) and 13 key informant interviews in 6 communities of NunatuKavut in Southern Labrador	Findings included that (w)holistic conceptualizations of health are essential to good oral health, achieving optimal oral health is prohibitive for Inuit communities, and community-engaged oral health service delivery is needed.

Mathu-Muju et al. [21]	Explore the experiences and opinions of First Nations families whose children had enrolled in the Children's Oral Health Initiative (COHI)	Interviews, *n* = 141, were completed in 13 communities. Six open-ended questions guided the interview process. Content analysis was used to code transcripts and identify themes	Findings were that local, community-based oral health prevention program needs to be further integrated into traditional aboriginal holistic models of wellness

Leck et al. [22]	Explores the rise and fall of the dental therapy profession in Canada and the resulting impact on Inuit and First Nations communities in terms of access to basic oral healthcare	A policy analysis was conducted using historical and policy documents	Factors identified included the following: First Nations communities are often small and scattered across Canada, frequently in rural or remote locations; unique cultural perspectives held by Inuit and First Nations people; blended responsibility for healthcare makes addressing equity issues less than straightforward as the division of federal and provincial powers creates a policy legacy that constrains future policy options

Lawrence et al. [5]	(i) Assess whether there were associations between oral health-related outcomes and self-reported racism and (ii) if they existed, whether associations between oral health-related outcomes and self-reported racism persisted after adjusting for significant covariates in our sample. Other objectives of the study were (i) to compare the prevalence of self-reported racism among the three countries collaborating on an early childhood caries preventive trial and (ii) to compare the findings with prevalence estimates reported in First Nations-governed national health surveys in Canada	“Baby Teeth Talk (BTT)” study, a community-based early childhood caries (ECC) randomized controlled trial, which is testing a multipronged behavioral and preventive intervention among 544 pregnant Canadian aboriginal women and their children living in urban and on-reserve communities in the provinces of Ontario and Manitoba	Findings included that one-third of participants experienced racism in the past year determined by the Measure of Indigenous Racism Experience and that racism experienced by aboriginal women can be a barrier to accessing dental-care services

Cidro et al. [23]	Describe how infant feeding practices, including breastfeeding, are a part of the larger maternal Indigenous knowledge transmission process that can aid in promoting healthy infants, including oral health	The Baby Teeth Talk study (BTT): a total of twenty interviews were held and four focus groups. The participants were primarily grandmothers and mostly great grandmothers, some of whom were former and current primary healthcare providers in various capacities, both in traditional health as in the biomedical field	Findings included the importance of understanding that cultural health traditions are essential for those working in oral public health capacities to ensure there is community acceptance of the interventions

Naidu et al. [24]	Explore oral health beliefs and practices and factors related to child oral health promotion in the Algonquin community of Rapid Lake, Quebec	Participants included children, parents, educators, healthcare workers, youth workers, and elders. Semistructured interviews were conducted with key informants. The following two focus group interviews were conducted: one with parents and one with school children	Findings included that a gap existed between oral health knowledge and oral health behaviors; challenges for oral health promotion included attitudes and beliefs, access, and priorities; parents needed to be further integrated into health promotion strategies

Prowse et al. [25]	Examine the knowledge and beliefs of parents and caregivers from four different cultural groups with respect to early childhood oral hygiene (ECOH) and early childhood caries (ECC)	A qualitative study design using focus groups was chosen to explore parent and caregiver views on ECOH and ECC	Challenges identified included uncooperative children, the cost and inability to purchase oral hygiene supplies, lack of time, difficulty in getting their children to see the dentist, previous negative experiences (they had been scared or hurt during previous dental encounters or feared needles), and lack of knowledge about the link between baby bottles at bedtime and dental decay

Blanchard et al. [26]	Analyzing oral health-related symptoms and behaviors, examined associations with other lifestyle factors, and sociodemographic variables to better understand the underlying and proximate determinants of poor oral health in these First Nation and Caucasian communities	First Nation and Caucasian participants completed a questionnaire on sociodemographic variables, oral health symptoms, and oral health-related behaviors as part of a broader cohort study comparing these ethnic groups for different chronic immune-mediated diseases	Findings included that lower levels of education, living rurally, and smoking were all related to poorer oral hygiene habits

Anonymous [27]	N/A	First Nations Oral Health Survey (FNOHS): the survey was released on September 27, 2012	The finding was that lack of access to regular dental-care results in poor oral health

Mejia et al. [28]	Overview of the oral health inequalities between Indigenous and non-Indigenous child and adult populations in the United States, Canada, Brazil, Australia, and New Zealand	Data from representative surveys were used	Factors identified were historical factors (isolation, discrimination, and alienation); culturally inappropriate oral health service provision, geographic factors, social determinants, and reliable national-level data

Pacey et al. [29]	Provide the prevalence and correlates of parental-reported oral health of Inuit preschoolers, with a focus on dietary and socioeconomic risk factors	Inuit preschool-aged children aged 3 to 5 years from 16 of Nunavut's 25 communities were randomly selected to participate in the Nunavut Inuit Child Health Survey conducted in 2007 and 2008	Findings included the likely importance of nutritional health education and better access to nutritious foods for promoting oral health

Leake [4]	Describe the oral health status and determinants among children 2–6 years of age in the Inuvik Region	In 2004–2005, 349 of 541 eligible, mostly preschool, children in the Inuvik Region in the Northwest Territories of Canada were examined clinically, and the parents or caregivers of 315 of these children were interviewed to measure their oral health status, and its impacts and determinants	Four determinants of oral health were protective factors: higher family incomes, community water fluoridation, and drinking milk and drinking fruit juices after the child began to walk

Schroth et al. [30]	Report findings from interviews with primary caregivers on their knowledge and attitudes toward preschool oral health and ECC from 4 communities in the province of Manitoba, Canada, that took part in an epidemiological study of early childhood dental health	Participation was restricted to those younger than 72 months of age. Children and their primary caregivers who participated in an institutional review board (IRB)-approved study of the prevalence of ECC in 4 Manitoba communities served as the sample for this report	Findings included that caregivers who believed that baby teeth are important were more likely to have children with better oral health (i.e., less decay) than those who thought otherwise, but also caregivers of children with ECC were more likely to disagree that dental decay could affect a child's overall health

**Table 3:** Challenges to the provision/utilization of oral healthcare (modified CAHS outline).

Major themes	Subthemes	Final codes
Individual characteristics	Age	Children/elderly
	Sex	Male or female
	Medical status	Pregnant
		Poor medical health
	Psychological status	Anxiety and fear of the dentist.
		Uncooperative children.
		Perceived inability to change oral health.
	Education	Limited education
	Employment	Employment and working conditions
	Oral health literacy	Limited oral health knowledge
	Habits	Smoking
	Lifestyle	Lack of healthy food, clean water, adequate sewage systems, electricity, or paved roads
		Lack of knowledge regarding healthy food choices and healthy infant feeding practices
		Lack of water fluoridation
		Social attitudes against healthy infant feeding practices (breastfeeding)
	Behaviors	Behaviors that do not support oral health
		Gap between oral health knowledge and oral health behaviors
	Historical background	Isolation, discrimination, and alienation

Affordability	Economic status	Lack of dental insurance
		Lack of affordability of healthy food
		High cost of dental services
		Low income
		Scarce housing
		Lack of affordability of oral hygiene supplies

Availability	Resources availability	Lack of human resources to support on-reserve oral healthcare
		Lack of funding to support community programs and facilities
		Many of these programs operate on uncertain year-to-year funding cycles

Accessibility	Location	Living in remote areas
		Lack of access to regular dental care
	Transportation	Lack of transportation to oral-care services

Accommodation	Meeting the needs and limitations of the client	Lack of financial incentive for the oral-care provider
		Lack of accurate and complete records of patients
		Lack of effective communication
		Language barrier (some elders do not speak English or French)

Acceptability	Patient-oral-care provider communication	Lack of understanding/integration of the (W) holistic conceptualization of health
		Lack of knowledge and skills and scope of practice
		Lack of training for the healthcare providers
		Lack of community engagement
		Racism
		Lack of trust/communication
		Ignore or undermine traditional medical practices, attitudes, and health knowledge

Public/government	Policy	Low priority for dental care
		Logistical aspects related to the administration, structure, and sustainability of oral health programs
		Lack of data availability from scientific research
		Budgetary constraints
		Division of federal and provincial powers create a policy legacy that constrains future policy options

## Data Availability

The data are included in the Materials and Methods section.
